# Information needs of patients with cancer: results from a large study in UK cancer centres

**DOI:** 10.1054/bjoc.2000.1573

**Published:** 2001-01-01

**Authors:** V Jenkins, L Fallowfield, J Saul

**Affiliations:** Department of Oncology, Royal Free and University College London Medical School, 48 Riding House Street, London, W1P 7PL, UK

**Keywords:** information needs, cancer, patients preferences

## Abstract

As part of a multi-centred study evaluating a communication skills training model for clinicians, we collected information preferences using an adaptation of Cassileth's Information Needs questionnaire from a heterogeneous sample of 2331 patients. Results showed that 87% (2027) wanted all possible information, both good and bad news and 98% (2203) preferred to know whether or not their illness was cancer. Cross tabulation of responses revealed no significant differences in information preferences for tumour site or treatment aims but did show an effect of age and sex. The few 58/440 (13.2%) patients who stated that in general they preferred to leave disclosure of details up to the doctor, tended to be older patients more than 70 years of age (chi square = 26.01, df = 2, *P*< 0.0001), although paradoxically they still wanted to know certain specific details. In comparison to men women preferred to know the specific name of the illness (chi square = 4.9, df = 1, *P*< 0.02) and what were all the possible treatments (chi square = 8.26, df = 1, *P*< 0.004). The results from this very large sample provide conclusive evidence that the vast majority of patients with cancer want a great deal of specific information concerning their illness and treatment. Failure to disclose such information on the grounds that significant numbers of patients prefer not to know is untenable. © 2001 Cancer Research Campaign http://www.bjcancer.com


British Journal of Cancer (2001) **84**(1), 48–51
© 2001 Cancer Research Campaign
doi: 10.1054/ bjoc.2000.1573, available online at http://www.idealibrary.com on IDEAL®

### Information needs of patients with cancer: results from a large study in UK cancer centres

**V Jenkins, L Fallowfield and J Saul**

Royal Free and University College London Medical School, Department of Oncology, 48 Riding House Street, London W1P 7PL, UK

**Summary** As part of a multi-centred study evaluating a communication skills training model for clinicians, we collected information preferences using an adaptation of Cassileth's Information Needs questionnaire from a heterogeneous sample of 2331 patients. Results showed that 87% (2027) wanted all possible information, both good and bad news and 98% (2203) preferred to know whether or not their illness was cancer. Cross tabulation of responses revealed no significant differences in information preferences for tumour site or treatment aims but did show an effect of age and sex. The few 58/440 (13.2%) patients who stated that in general they preferred to leave disclosure of details up to the doctor, tended to be older patients more than 70 years of age (chi square = 26.01, df = 2, $P < 0.0001$), although paradoxically they still wanted to know certain specific details. In comparison to men women preferred to know the specific name of the illness (chi square = 4.9, df = 1, $P < 0.02$) and what were all the possible treatments (chi square = 8.26, df = 1, $P < 0.004$). The results from this very large sample provide conclusive evidence that the vast majority of patients with cancer want a great deal of specific information concerning their illness and treatment. Failure to disclose such information on the grounds that significant numbers of patients prefer not to know is untenable. © 2001 Cancer Research Campaign http://www.bjcancer.com

**Keywords**: information needs; cancer; patients preferences

Despite the publication of several quantitative studies, (Fallowfield et al, 1994; Meredith et al, 1996; National Cancer Alliance, 1996) some health professionals remain concerned about the amount and type of information to give to a patient with cancer. Would for example, the elderly widow with cancer have similar information needs to the 50-year-old professional woman with cancer? Should clinicians edit the information that they give based on intuition and past experience or should they do so only according to patients' stated preferences?

We know that clinicians tend to underestimate the amount of information that patients require (Fallowfield et al, 1994; Degner et al, 1997) and while fewer these days are reluctant to use the word cancer, many still believe that disclosure should only be made to those patients who actively seek it. Unfortunately unless invited to ask directly, patients rarely raise important questions during a consultation. Many patients assume that the doctor would have told them everything relevant, others worry that they will appear foolish if they reveal their ignorance by asking questions, and some feel that they have already taken up too much of the busy doctor's time (Fallowfield and Jenkins, 1999). At least two small studies of non-representative groups of patients (Cassileth et al, 1980; Fallowfield et al, 1994) have shown that patients' information needs are substantial. This finding was also validated in a small, but carefully stratified, more representative sample of 250 patients with cancer (Meredith et al, 1996). In contrast, a recently published qualitative study of 17 patients, suggested that some may prefer to avoid disease related information at different stages during the illness (Leydon et al, 2000); although precise methodological detail is lacking about such basic issues as the representativeness of the sample which hampers interpretation of the study.

We present results from a survey of a heterogeneous sample of 2331 patients attending out-patient departments throughout the United Kingdom for consultations about cancer and its treatment, that unequivocally answers the question of how much information patients prefer to have from their doctors.

*Received 9 June 2000*
*Revised 16 September 2000*
*Accepted 11 October 2000*

*Correspondence to:* V Jenkins

### PARTICIPANTS AND METHODS

As part of a study evaluating a communication skills training model for clinicians, we collected information preferences from a heterogeneous sample of patients attending out-patient clinics within the UK. 34 hospitals were involved in the study, including large teaching hospital cancer centres and smaller district general hospitals. MREC and LREC was granted for the patients' assessments. Inclusion criteria were broad and comprised adults who were about to see a medical, clinical or surgical oncologist for a consultation about their cancer diagnosis, treatment, prognosis or tests and routine follow-up visits.

2681 patients were approached by a member of the research team whilst waiting in out-patient clinics to see the doctor and given an information sheet explaining the study. 85% (2331) gave written informed consent to participate. The most common reasons given for non-participation were a lack of English, poor eye-sight and concerned relatives. Table 1 lists the tumour site details, aim of treatment and patient characteristics. The questionnaire is one that is widely used throughout the UK and US in previously published studies (Fallowfield et al, 1994; Meredith et al, 1996). It consists of two parts; in part one, patients were asked to choose a statement that best described their general attitudes

**Table 1** Background details of patients ($n$ = 2331)

| Characteristics | Sample |
|---|---|
| Age (years) | |
| <=30 | 113 (4.8%) |
| 31–50 | 580 (24.9%) |
| 51–70 | 1081 (46.4%) |
| >70 | 440 (18.9%) |
| Unknown | 117 (5%) |
| Sex | |
| Men | 892 (38.3%) |
| Women | 1439 (61.7%) |
| Marital status | |
| Partner | 1590 (68.2%) |
| No partner | 612 (26.3%) |
| Unknown | 129 (5.5%) |
| Tumour site | |
| Breast | 585 (25.1%) |
| Gastro-intestinal/colorectal | 418 (17.9%) |
| Haematological | 181 (7.8%) |
| Lung | 164 (7.0%) |
| Gynaecological | 147 (6.3%) |
| Urological | 145 (6.2%) |
| Skin | 80 (3.4%) |
| CNS | 64 (2.7%) |
| Head & Neck | 57 (2.4%) |
| Unknown Primary | 164 (7.0%) |
| Other | 326 (14.0%) |
| Type of treatment | |
| Curative | 940 (40.3%) |
| Palliative | 841 (36.1%) |
| Remission | 215 (9.2%) |
| Not Specified | 335 (14.4%) |

and preferences about information concerning their illness. Part two consisted of a list of 7 different more specific kinds of information about illness and treatment and patients were asked to indicate whether they 1) had an absolute need, 2) would like to have or 3) would prefer not to have that information. Answers to the questions were cross-tabulated according to the patient's age, sex, aim of treatment and cancer site. Statistical significance was assessed using the chi square test.

### RESULTS

Of the 2331 patients who participated in the study, 940 (40%) were receiving curative treatment, 841 (36%) palliative treatment, 215 (9%) were in remission and for 335 (14%) treatment intent was still uncertain.

### Part 1

In response to the first question, on patients' general attitudes to information, 2027 (87%) preferred to have as much information as possible, both good and bad. 125 (5.4%) preferred to have additional information only if it was good news and 179 (7.7%) preferred to leave it up to the doctor.

### Part 2

Table 2 shows an overwhelming need by patients for specific information concerning different aspects of illness and treatment. Only 42/2231 (1.9%) did not want to know if they had cancer.

### CROSS TABULATION OF RESPONSES

### Age

There was a significant association between age and the amount of information required by the patient. Although a large proportion of patients over 70 years of age wanted as much information as possible (81%), significantly more of the older group preferred to leave details up to the doctor (13% 58/440 v 6.5% 110/1774; chi square = 26.01, df = 2, $P < 0.0001$). In addition, there were significant differences in response to the specific questions as shown in Table 2, although it should be emphasized that the majority of the older patients did want detailed information.

### Sex

Although the majority of patients of both sexes wanted specific details of information, there were two areas where the younger women (70 years and under) were significantly different. They preferred to know the name of the illness, (974/1056 [92%] v 588/660 [89%], chi square = 4.9, $P < 0.02$) and all the possible available treatments 1021/1059 [96%] v 621/665 [93]%, chi square = 8.26, $P < 0.004$).

### Aim of treatment

There were no differences in specific information needs between those patients receiving curative or palliative treatment or for those who were in remission.

### Cancer site

There were no differences in information needs between the different cancer sites. This was despite the fact that the largest cancer site in the sample was women with breast cancer.

### The specific information needs of patients who want to leave details up to the doctor and 'good news' patients

Although a small percentage of the sample stated in part one that they preferred to leave everything up to the doctor or wanted only good news (7.7% and 5.4% respectively), when prompted by the items in part two of the questionnaire, the majority of patients in both groups expressed a need for specific information concerning illness and treatment. Table 3 shows the responses of all 3 groups with the data from 'absolutely need' and 'would like to have' collapsed into one category.

### DISCUSSION

The results from this large survey support previous research that most patients want as much information as possible about treatments and illness. An extremely important finding was that 98% of patients needed to know whether the illness was cancer. This observation should convince health professionals that they should not withhold the truth about diagnosis on the grounds that many patients prefer not to know. Furthermore 95% wished to know what their chances of cure were, so information about prognosis should not be avoided either (Fallowfield et al, 1998). Our findings contrast with a recent qualitative study of 17 patients which suggested that patients' strategies for coping with cancer suppress their wish for, and efforts to obtain information at different stages of their illness. Qualitative

© 2001 Cancer Research Campaign 

**Table 2** Responses of 2331 patients to specific information needs (*n* and valid %)

| Question | Total Sample | | | ⩽ 70 years of age | > 70 years of age | |
|---|---|---|---|---|---|---|
| | Absolutely need | Would like to have | Do not want to have | Absolutely need and would like | Absolutely need and would like | chi square *P* value |
| What the specific medical name of the illness is. | 801 (35.6%) | 1193 (53.1%) | 254 (11.3%) | 1562/1716 (91%) | 334/419 (79.7%) | 43.34 $P = 0.0001$ |
| Whether or not it is cancer. | 1348 (60%) | 855 (38.1%) | 42 (1.9%) | 1684/1711 (98.4%) | 408/421 (96.9%) | 4.18 $P = 0.04$ |
| When you are having treatment what the week by week progress is. | 876 (39.3%) | 1148 (51.5%) | 203 (9.1%) | 1566/1701 (92.1%) | 359/416 (86.3%) | 13.47 $P = 0.0002$ |
| What the chances of cure are. | 1195 (53.9%) | 920 (41.5%) | 104 (4.7%) | 1626/1696 (95.9%) | 384/412 (93.2%) | 5.32 $P = 0.021$ |
| What all the possible treatments are. | 1223 (54.2%) | 897 (39.8%) | 135 (6%) | 1642/1724 (95.2%) | 373/418 (89.2%) | 21.78 $P = 0.0001$ |
| What all the possible side effects of treatment are. | 1363 (60%) | 840 (37.1%) | 62 (2.8%) | 1692/1729 (97.9%) | 403/422 (95.5%) | 7.46 $P = 0.006$ |
| How the treatment works to treat the illness. | 1027 (45.6%) | 1042 (46.3%) | 183 (8.1%) | 1612/1719 (93.8%) | 355/420 (84.5%) | 39.06 $P = 0.0001$ |

**Table 3** Responses to specific information needs as a function of general preference for information

| | All information | Only good news | Leave up to Dr. |
|---|---|---|---|
| Question | Absolutely need and would like | Absolutely need and would like | Absolutely need and would like |
| Specific name of the illness | 1822/1962 (93%) | 79/118 (67%) | 93/168 (55%) |
| Whether or not it is cancer | 1930/1961 (98%) | 108/117 (92%) | 145/167 (87%) |
| Week by week progress | 1839/1949 (94%) | 82/116 (71%) | 103/162 (64%) |
| Chances of cure | 1909/1943 (98%) | 88/115 (77%) | 118/161 (73%) |
| All the possible treatments | 1919/1969 (97%) | 92/119 (77%) | 109/167 (65%) |
| All the possible side effects | 1956/1975 (99%) | 112/121 (93%) | 135/169 (80%) |
| How the treatment works | 1887/1965 (96%) | 82/121 (68%) | 100/166 (60%) |

methods can provide a different perspective but they must be rigorous (Mayes and Pope, 1996). It would be wrong for health professionals to alter their practice of information giving based on such limited results. The article had so few details about the methodology utilized for sample selection and the manner in which data were collected and analysed to permit independent judgement about the conclusions. Instead of still questioning the need for giving more information we need to be developing ways to provide adequate information in a flexible and sensitive manner.

The avoidance of information often stems from myths and misunderstandings about the disease which doctors could correct with clearer explanations and thus alleviate distress. The majority of patients want to know, as we have shown but may be afraid to ask. Cross-sectional analysis of our data showed no evidence for different information needs whether patients were awaiting diagnosis, having radical treatment, were in remission or being treated palliatively. However, to ensure that patients' true views and preferences are met throughout the disease trajectory, doctors need to adopt flexible policies of regularly checking information needs more directly with their patients during their consultations.

The attitudes and beliefs held by patients and doctors clearly influence the consultation. The notion that the older patient prefers the doctor to determine how much information to provide is only weakly upheld by the study. Although significantly more of the older (i.e., those over 70 years) patients indicated a preference to leave details up to the doctor, most (98%) still wanted specific information about treatment and side effects, especially whether or not they had cancer. Negative stereotypes of the elderly are common among health care professionals (Greene et al, 1986). If clinicians assume that there is an increase in passivity and helplessness in the elderly patient, then it is more likely that these negative aspects will prevail in the consultation. This leads to a doctor-centred rather than patient-centred interaction, with the doctor in control of information giving.

Many older people still have a deferential attitude towards doctors, particularly when meeting them in a medical setting; thus our findings demonstrate the need for doctors to actively encourage such patients to ask questions. These patients may have grown up in a culture where it was considered impolite to question or ask further information from a 'busy' specialist. It would be surprising to still find this attitude in 10 or 20 years time, as medical information becomes more accessible to the general public. Clinicians have already commented on the increase in the number of 'internet patients' attending clinics, who are sometimes more informed about new treatments than the doctor (Thompson, 1999).

Recent literature repeatedly states that patients, whatever age, want to be kept well informed about their illness. The findings are similar for the emergency patient in the USA (Davis et al, 1999), the pre-operative patient in Australia (Farnhill and Inglis, 1994) the cancer patient in Hong Kong (Fielding and Hung, 1995) and the woman with breast cancer in Liverpool (Luker et al, 1996) or Canada (Degner et al, 1997). However, although older patients have a high desire for information, some data suggest that they have less desire for participation

 *© 2001 Cancer Research Campaign*

in decisions about treatments and management (Charles et al, 1998). Some clinicians have difficulty separating decision making from a need for information. Although some older people may want the doctor to make decisions about management, they still require information about the reasons for decisions affecting their care.

Age remained a significant factor when examining whether men and women wanted the same detailed information. Younger women had a greater need for information about all the possible available treatments, a finding similar to that in a previously published study of a small but representative sample of patients in the West of Scotland (Meredith et al, 1996). More younger women also preferred to know the specific name of the illness than men of the same age group. Women as a group are more active in seeking information about health and illness, for example CancerBACUP reported an excess of female enquirers to their information helpline (Boudioni et al, 1999) and in a recent study, 27% of women compared with 15% of men accessed the internet for health information at least once a week (Eysenbach et al, 1999).

Information about treatment options, prognosis and side-effects was considered particularly important by the patients surveyed by the National Cancer Alliance (1996). The majority of patients indicated that they wanted to know the truth and to be offered information in a language that they could understand. The Calman-Hine Report (1995) recommends that the views and preferences of patients, families and carers should be taken into account and patients given clear, understandable information. It has been shown that providing patients with information according to their own agendas facilitates psychological adaptation to illness and treatment (Fallowfield et al, 1990). One way to achieve this aim is through good communication. Following the Calman-Hine report into cancer services, attention to communication and information giving is an explicit recommendation in the NHS Executive guidelines yet it is an area that is often under-resourced. In addition there is anecdotal evidence from hospitals and medical defence organizations that suggests poor communication and inadequate information can result in complaints and litigation. Although discussions about diagnosis, prognosis and clinical trials in time pressured clinics are difficult, health care professionals can learn effective communication skills to assist them with these tasks (Maguire et al, 1996; Razavi and Delvaus, 1997; Wilkinson et al, 1998). It has been shown that doctors engage in more patient-centred behaviour following training which means that they are more flexible and responsive to patients' needs (Fallowfield et al, 1998).

The results from this very large sample support previous findings and is much more representative of cancer patients as a whole. It shows that most patients want as much information as possible. Even though patients may indicate a general preference for good news only or to leave details up to the doctor, most when cued by the questionnaire about different aspects of disease and treatment, still want very specific information. Failure to disclose information out of a belief that patients prefer not to know is untenable and prior knowledge of patients' specific information needs might assist doctors in tailoring their consultations to meet patients' actual not inferred requirements.

### ACKNOWLEDGEMENTS

We would like to thank all those who participated and the Cancer Research Campaign for funding the study.

Contributors: Lesley Fallowfield had the original idea for the study and Val Jenkins, Lesley Fallowfield and Jacky Saul each contributed to data analysis and writing the paper. Tony Duffy, Anna Fair, Phillipa Jones, Sarah Ford Angela Hall and Denise Ratcliffe together with the authors were involved in the data collection for the study.

### REFERENCES

Audit commission (1993) *What seems to be the matter? Communication between hospitals and patients*. London:HMSO (NHS Report No 12)

Boudioni M, McPherson K, Mossman J, et al. (1999) An analysis of first-time enquirers to the CancerBACUP information service: variations with cancer site, demographic status and geographical location. *Br J Cancer* **79**(1): 38–145

Calman F and Hine D (1995) *Expert Advisory Group on Cancer. A Policy Framework for Commissioning Cancer Services*. London: Department of Health

Cassileth B, Zupkis R and Sutton-Smith Kea (1980) Information and participation preferences among cancer patients. *Ann Intern Med* **92**: 832–836

Charles C, Redko C, Whelan T, Gafni A and Reyno L (1998) Nothing is no choice: lay constructions of treatment decision making among women with breast cancer. *Sociol Health and Illness* **20**: 71–95

Davis MA, Hoffman JR and Hsu J (1996) Impact of patient acuity on preference for information and autonomy in decision making. *Acad Emerg Med* **6**(8): 781–5

Degner LF, Kristjanson LJ, Bowman D et al. (1997) Information needs and decisional preferences in women with breast cancer. *JAMA* **277**(18): 1485–1492

Eysenbach G, Sa ER and Diepgen TL (1999) Shopping around the internet today and tomorrow: towards the millennium of cybermedicine. *Br Med J* **319**(13 Nov): 1–5

Fallowfield LJ (1997) Truth sometimes hurts but deceit hurts more. *Communication with the Cancer Patient* **809**: Annals of the New York Academy of Sciences 525–535

Fallowfield L and Jenkins V (1999) Effective communication skills are the key to good cancer care. *Eur J Can* **35** (11): 1592–1597

Fallowfield L, Hall A, Maguire G and Baum M (1990) Psychological outcomes of different treatment policies in women with early breast cancer outside a clinical trial. *Br Med J* **301**: (575–580)

Fallowfield L, Ford S and Lewis S (1994) No news is not good news: Information preferences of patients with cancer. *Psycho-oncology* **4**: 197–202

Fallowfield L, Lipkin M and Hall A (1998) Teaching senior oncologists communication skills: results from phase I of a comprehensive longitudinal program in the United Kingdom. *J Clin Oncol* **16**(5): 1961–8

Farnill D, Inglis S (1994) Patients' desire for information about anaesthesia: Australian attitudes. *Anaesthesia* **49**(2): 162–4

Fielding R and Hung J (1995) Preference for information and treatment-decision making involvement in cancer care among a Hong Kong Chinese population. *Psycho-Oncology* **5**: 321–329

Greene MG, Adelman R, Charon R and Hoffman S (1986) Ageism in the medical encounter: An exploratory study of the language and behaviour of doctors with their old and young patients. *Language and communication* **6**: 113–124

Leydon G, Boulton M, Moynihan C, et al. (2000) Cancer patients' information needs and information seeking behaviour: in depth interview study. *Br Med J* **320**: 909–13

Luker KA, Beaver K, Leinster SJ and Owens RG (1996) Information needs and sources of information for women with breast cancer: a follow-up study. *J Adv Nurs* **23**(3): 487–95

Maguire P, Booth K, Elliott C and Jones B (1996) Helping health professionals involved in cancer care acquire key interviewing skills – the impact of workshops. *Eur J of Can* **32A**(9): 1486–1489

Mayes N and Pope C (1996) Rigour and qualitative research. In: Mayes N and Pope C (eds) *Qualitative Research in Health Care* London: British Medical Journal Publishing Group

Meredith C, Symonds P, Webster L, et al. (1996) Information needs of cancer patients in West Scotland; cross sectional survey of patients' views. *Br Med J* **313**: 724–726

National Cancer Alliance (1996) Patient – centred cancer services? what patients say. Oxford: 6 National Cancer Alliance

NHS Executive: (1998) Clinical Guidelines: using clinical guidelines to improve patient care in the NHS. Leeds: NHS Executive

Razavi D and Delvaus N (1997) Communication skills and psychological training in oncology. *Eur J Can* **33** (Suppl 6): S15–S21

Thompson C (1999) Cybermedicine. *Br Med J* **319** (720): 1294

Wilkinson S, Roberts A and Aldridge J (1998) Nurse-patient communication in palliative care: an evaluation of a communication skills programme. *Palliative Med* **12**: 13–22

*© 2001 Cancer Research Campaign* 
